# Role of Src family kinases Fyn and Lyn in arenavirus infection

**DOI:** 10.1128/jvi.00241-26

**Published:** 2026-03-31

**Authors:** Wenting Mao, Minmin Zhou, Cheng Peng, Yi Wan, Li Li, Jialing Hu, Yang Liu, Yuan Peng, Chao Shan, Fei Deng, Zhiming Yuan, Wei Wang

**Affiliations:** 1 State Key Laboratory of Virology and Biosafety, Wuhan Institute of Virology, Center for Biosafety Mega-Science, Chinese Academy of Sciences 74614 , Wuhan, China; 2 University of the Chinese Academy of Sciences, Beijing, China; 3 Department of Microbiology, School of Clinical Medicine, Li Ka Shing Faculty of Medicine, The University of Hong Kong, Pokfulam, China; 4 College of Life and Health Sciences, Wuhan University of Science and Technology, Wuhan, China; University of Kentucky College of Medicine, Lexington, Kentucky, USA

**Keywords:** LASV, LCMV, Src family kinases (SFKs), phosphorylation, saracatinib

## Abstract

Arenaviruses encompass a diverse group of infectious agents, some of which, like Lassa virus (LASV), pose significant threats to global public health due to their acute virulence, while others, such as lymphocytic choriomeningitis virus (LCMV), are less pathogenic but still clinically relevant. Both Old World arenaviruses LASV and LCMV exploit host tyrosine kinase signaling to establish infection, though the molecular mechanisms remain incompletely understood. In this study, phosphorylated receptor tyrosine kinase antibody array screening revealed that recombinant LASV (rLCMV-LASV GP) infection specifically activates Src family kinases (SFKs), Fyn and Lyn, with their phosphorylation levels markedly elevated during early infection. Subsequent co-immunoprecipitation assays confirmed rLCMV-LASV GP-induced tyrosine phosphorylation of Fyn and Lyn activation. RNAi experiments demonstrated that knockdown of Fyn/Lyn significantly suppressed rLCMV-LASV GP and LCMV nucleocapsid protein expression, viral RNA synthesis, and viral production, highlighting the critical role of SFKs in viral infection. Further investigations showed that saracatinib, an Src inhibitor, exhibited broad-spectrum antiviral activity against LCMV strains, as well as the authentic LASV strain Lassa_HX strain. *In vivo* studies revealed that saracatinib substantially reduced viral loads in splenic and hepatic tissues and alleviated infection-associated histopathological damage. These findings identify Src family kinase Fyn/Lyn as novel host targets for arenavirus and provide new targets of prevention and treatment for arenaviral infection.

**IMPORTANCE:**

Arenaviruses, including the highly pathogenic Lassa virus (LASV) and clinically relevant lymphocytic choriomeningitis virus (LCMV), pose severe global health threats due to limited therapeutic options. This study unveils a critical host-pathogen interaction mechanism by which Src family kinases (SFKs) Fyn and Lyn facilitate LASV and LCMV infection. Through phosphorylation profiling and functional validation, we establish that viral activation of these kinases drives arenavirus infection. Moreover, the FDA-approved Src inhibitor saracatinib exhibits broad-spectrum efficacy, suppressing LCMV strains as well as the authentic LASV *in vitro*, while reducing LCMV viral loads and histopathology *in vivo*. These findings redefine tyrosine kinases as host targets, offering a novel host-directed antiviral strategy by repurposing existing clinical compounds.

## INTRODUCTION

Arenaviruses comprise a diverse genus of enveloped, bisegmented negative-sense single-stranded RNA viruses (1). Among them, Lassa virus (LASV), associated with lethal hemorrhagic fever, and clinically relevant members like lymphocytic choriomeningitis virus (LCMV) collectively contribute to substantial global healthcare burdens (2). Despite their clinical impact, management of severe cases remains limited to supportive care, highlighting an urgent need to identify effective antiviral drugs and therapeutic targets.

Viral entry into host cells begins with the interaction of viral particles with host cell membrane surface proteins, a critical early event determining the outcome of the viral infection cycle. For arenaviruses, while several well-characterized cellular receptors (α-dystroglycan, CD164, and transferrin receptor 1) are well-established mediators of viral entry, emerging evidence indicates that numerous other host proteins also regulate arenavirus infection (3–6). Among these, specific protein tyrosine kinases (PTKs) serve as key modulators: TAM family kinases (Tyro3, Axl, and Mer) and T cell immunoglobulin and mucin domain-containing protein 1 (TIM-1) have been experimentally validated to promote arenavirus entry, thus underscoring PTKs’ central role in facilitating viral host cell invasion (7–11). Src family kinases (SFKs), a major subfamily of nonreceptor PTKs, are membrane-associated signaling molecules defined by the absence of transmembrane and extracellular domains (12–14). SFKs orchestrate diverse cellular processes by activating context-dependent downstream signal transduction cascades, and they have been demonstrated to play pivotal roles in the entry and replication of various DNA and RNA viruses, including scale drop disease virus (SDDV), human immunodeficiency virus type 1 (HIV-1), and encephalomyocarditis virus (EMCV) (15–19).

However, the role of SFKs in regulating arenavirus infection remains incompletely defined. Herein, we used a phosphorylated protein tyrosine kinase antibody array to identify kinases activated during recombinant LASV infection and characterized the phosphorylation of several SFKs involved in LASV entry. To further confirm the conservation of this mechanism across arenaviruses, we extended these studies to the prototypic arenavirus LCMV. Moreover, given the broad regulatory roles of SFKs in viral infection, we also investigated the impact of clinically approved kinase inhibitors on arenavirus infection, with the aim of exploring their potential as antiviral targets.

## RESULTS

### Src family kinases activated in rLCMV-LASV GP-infected cells

**Fig 1:**
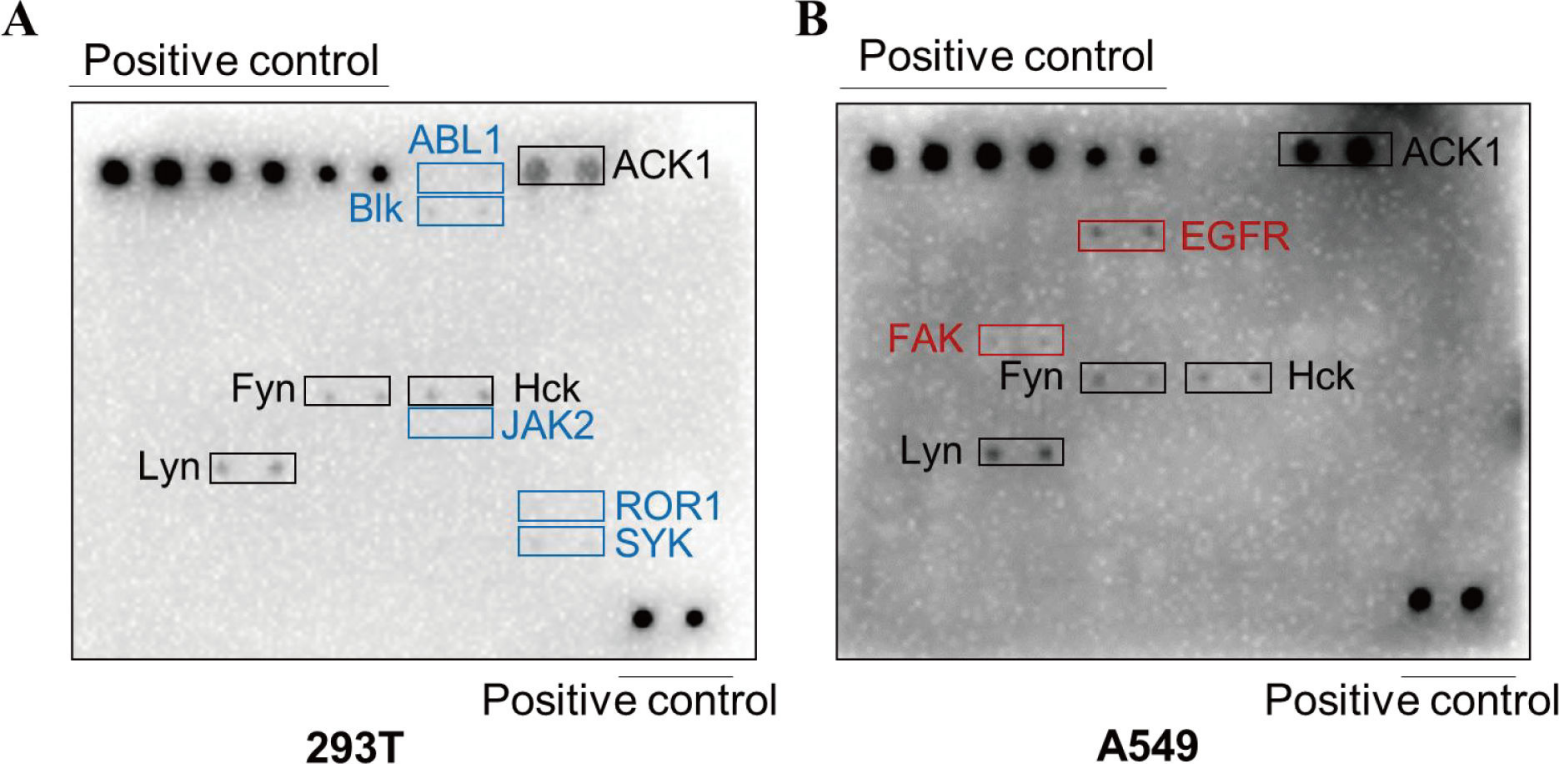
Differential relative protein expression profiles of human tyrosine kinase phosphorylation antibody array following viral infection. Phosphotyrosine kinase activation patterns were observed in 293T cells (**A**) and A549 cells (**B**) post-infection with rLCMV-LASV GP. The 293T and A549 cells were starved of serum for 24 h prior to infection with rLCMV-LASV GP at an MOI of 10. The mixed samples were first incubated on ice for 1 h and then incubated at 37°C for 15 min to promote viral entry. The cells were then collected, and the phosphorylation of 71 kinases in cell lysates was measured. Blue labels, the kinases activated only in 293T cells; red labels, the kinases activated only in A549 cells; black labels, the kinases activated in both cell lines.

To identify rLCMV-LASV GP-induced tyrosine kinase phosphorylation, a recombinant Lassa virus expressing the LASV glycoprotein (rLCMV-LASV GP) was constructed to perform a screening of the phosphorylated human receptor tyrosine kinase antibody array. The results demonstrated that rLCMV-LASV GP infection in 293T and A549 cells induced distinct activation patterns of receptor tyrosine kinases. In 293T cells, phosphorylation of ABL1, Ack1, Blk, Fyn, Lyn, Hck, JAK2, ROR1, and SYK kinases was observed, while in A549 cells, phosphorylation of Ack1, EGFR, FAK, Fyn, Hck, and Lyn kinases was detected. The phosphorylation of Ack1, Hck, Fyn, and Lyn kinases was found in both 293T and A549 cells after rLCMV-LASV GP infection. Among the activated kinases, Fyn, Lyn, and Hck belong to the Src family kinases (Fig. S1). The positive controls located in the upper left and lower right corners of the membrane were activated, validating the reliability of these experimental findings (Fig. 1).

**Fig 2:**
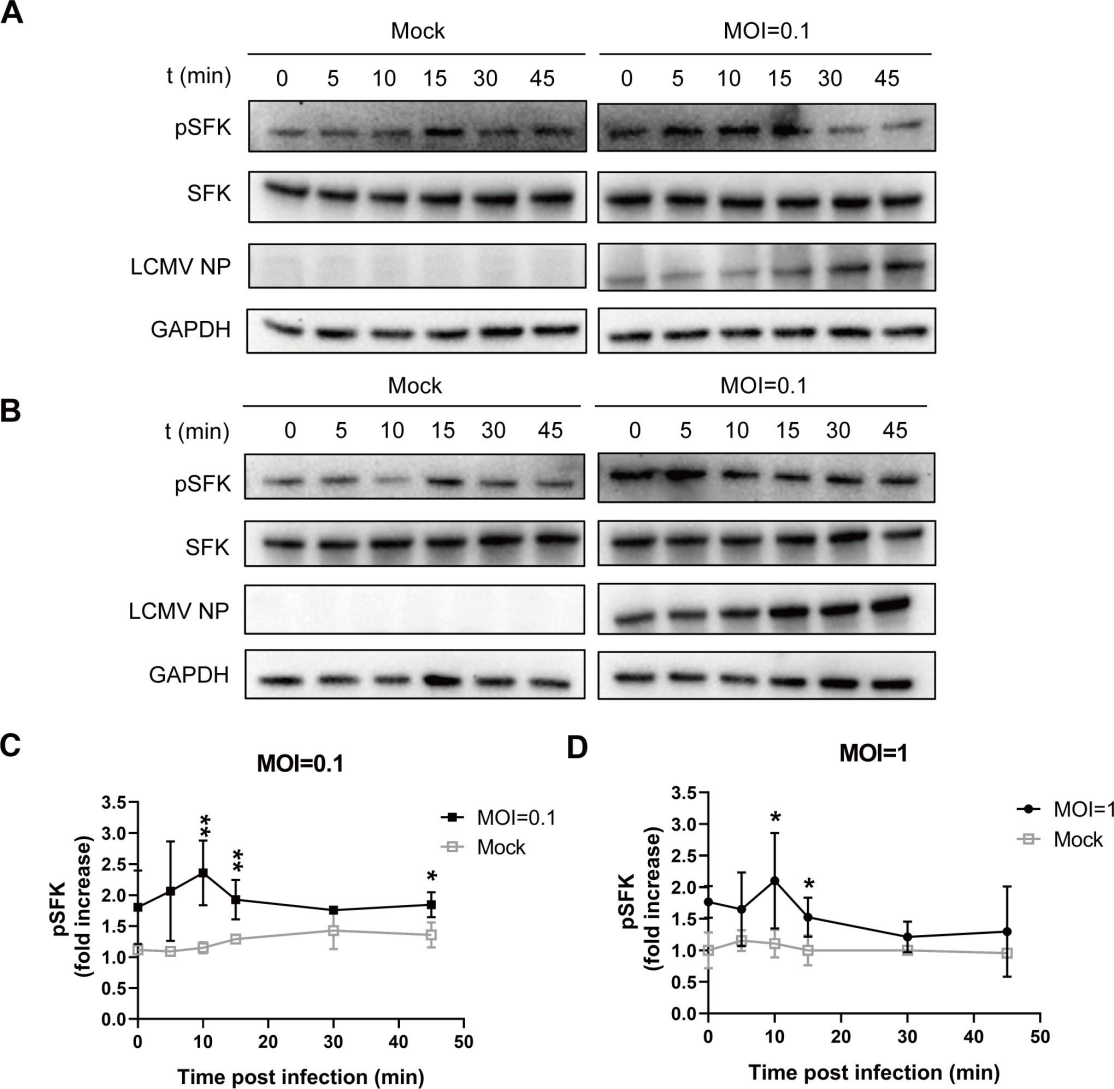
Activation of Src family kinases in response to rLCMV-LASV GP infection. A549 cells were infected with rLCMV-LASV GP at MOI = 0.1 (**A**) and MOI = 1 (**B**). After viral attachment to the cell surface on ice for 1 h, cells were transferred to a 37°C incubator to allow viral entry. Samples were harvested at 0, 5, 10, 15, 30, and 45 min post-viral entry initiation and immunoblotted with antibodies against phosphorylated SFKs, total SFKs, and anti-rLCMV-LASV NP antibodies to detect virions. GAPDH levels were measured as loading control. (**C and D**) Quantitative analyses of fold change of pSFK/total SFK of each sample compared to mock-infected cells set at 1. Data represent mean±SD of triplicate experiments. Significance relative to mock in panels C and D was determined by *t*-test; *P < 0.05; **P < 0.01.

To refine the conditions for rLCMV-LASV GP-induced tyrosine kinase phosphorylation, we varied infection time points and multiplicity of infection (MOI) for phosphorylation detection. Given the absence of specific phosphoantibodies for Fyn, Lyn, and Hck, viral kinase activation was validated using a pan-phospho-Src family kinases (pSFKs) antibody targeting the conserved autophosphorylation motif (Tyr416). Western blot (WB) analysis showed that the active phosphorylation level of SFKs (Tyr416) was generally higher in infected cells than in the mock group. At 0 min post-infection (mpi) in the infected group, the cells had been incubated with the virus at 4°C for 1 h. The elevated values observed in infected cells compared with the mock group at this time point reflected the early activation of SFKs triggered by virus-cell contact. A significant increase in pSFKs was observed within 10–15 min after rLCMV-LASV GP infection compared to the mock group (Fig. 2A). Similarly, significant phosphorylation of the SFKs was induced under MOI of 0.1 and 1 (Fig. 2B). Moreover, grayscale analysis of the phospho-SFK/total-SFK ratio revealed that kinase phosphorylation peaked 10 min post-infection (Fig. 2C and D). Overall, the Src signaling pathway may be involved in rLCMV-LASV GP infection.

### Knocking down the Fyn and Lyn kinases inhibited the infection of rLCMV-LASV GP

Screening of activated receptor tyrosine kinase antibody array revealed that the phosphorylation of four kinases, Ack1, Hck, Fyn, and Lyn, was observed in rLCMV-LASV GP-infected A549 and 293T cells. To investigate the role of four kinases, we employed an RNAi strategy for knocking down kinase genes. Three independent siRNA pairs for each kinase gene were designed to ensure target specificity. Viral infection efficiency was assessed through nucleoprotein (NP) quantification and plaque assays after kinase knockdown.

**Fig 3:**
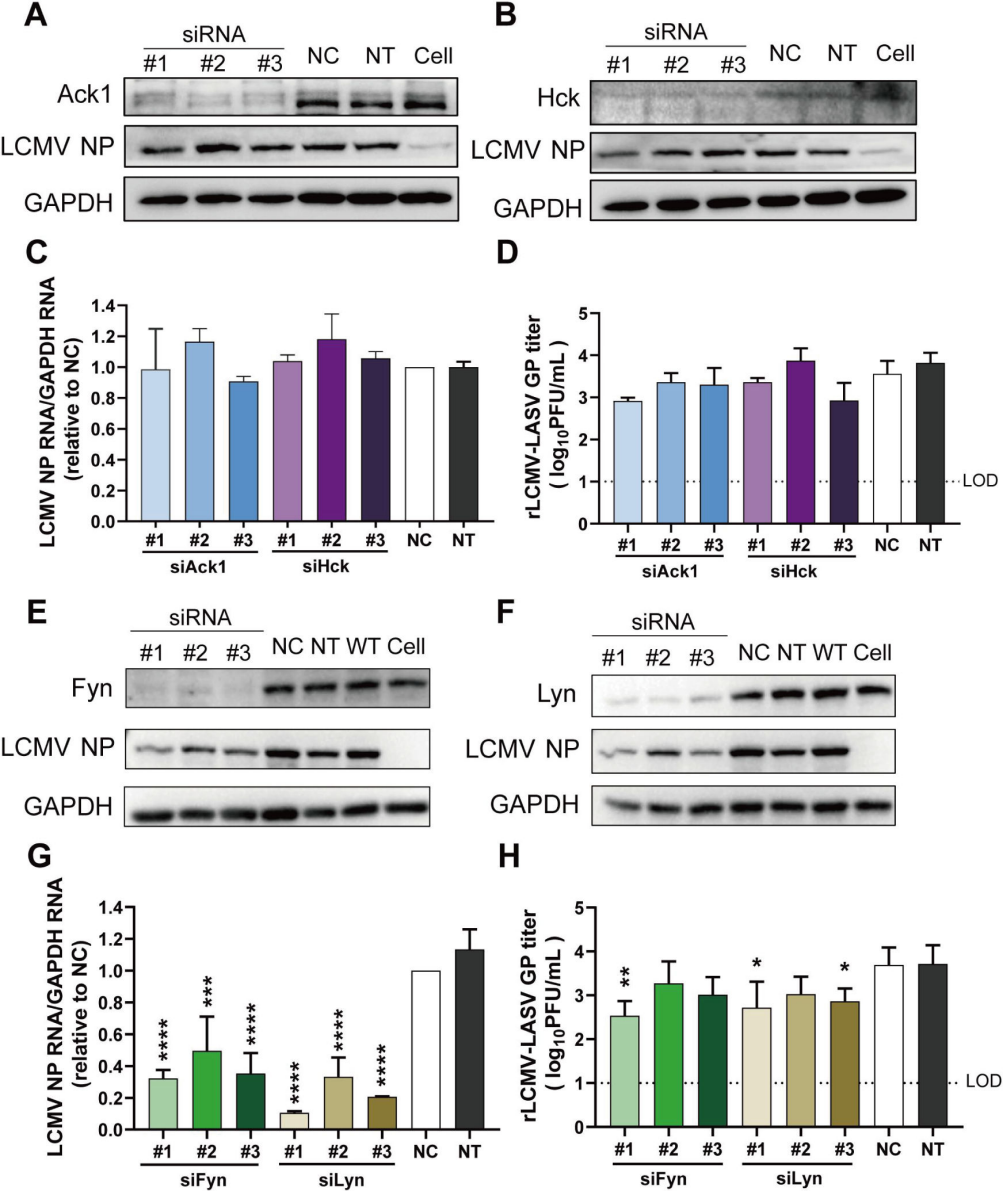
The effect of Ack1/Hck or Fyn/Lyn knockdown on rLCMV-LASV GP infection. Immunoblot analysis of viral protein expression in cells with Ack1 (**A**) or Hck (**B**) knockdown, infected with rLCMV-LASV GP. After siRNA transfection for 24 h, the cells were infected with rLCMV-LASV GP (MOI = 0.1) for 24 h. And then the supernatant was collected for plaque assay, and the cells were harvested for WB and viral RNA copy detection. (**C**) Quantification of viral mRNA expression relative to GAPDH by qRT-PCR assay. (**D**) Viral plaque assay for rLCMV-LASV GP production. (**E, F**) Immunoblot profiling of viral protein levels with Fyn or Lyn knockdown in rLCMV-LASV GP-infected cells. (**G**) qRT-PCR quantification of viral mRNA expression. (**H**) Viral plaque assay for rLCMV-LASV GP production across Fyn/Lyn knockdown. The significant differences were analyzed compared with the NC group. NC, negative control; NT, DEPC-treatment (DEPC-treated water) control; WT, viral infection group; cell, non-infection group; LOD, limit of detection. Data shown are expressed as mean±SD of three independent experiments. Significance was analyzed by one-way ANOVA; *P < 0.05, **P < 0.01, ***P < 0.001, and ****P < 0.0001.

Quantitative immunoblot analysis of NP expression across three biological replicates revealed that knockdown of endogenous Ack1/Hck did not result in significant changes in rLCMV-LASV GP NP expression levels (Fig. 3A and B). Plaque assays of cell culture supernatants revealed no significant changes in viral titers after Ack1 and Hck knockdown (Fig. 3C and D), indicating that Ack1/Hck kinases do not have a striking correlation with rLCMV-LASV GP infection.

In contrast, transfection with multiple pairs of siRNAs targeting Fyn and Lyn resulted in a significant downregulation of viral infection. The siRNAs effectively knocked down endogenous Fyn and Lyn expression, leading to a marked decrease in rLCMV-LASV GP NP protein levels (Fig. 3E and F). qRT-PCR and plaque assays revealed that knockdown of Fyn and Lyn dramatically reduced viral mRNA synthesis. Similarly, the plaque assay results showed that knockdown of Fyn and Lyn significantly reduced the production of progeny virus in the supernatant (Fig. 3G and H), demonstrating that Fyn and Lyn are protein tyrosine kinases that promoted rLCMV-LASV GP infection.

### Phosphorylation of both Fyn and Lyn kinases in rLCMV-LASV GP and LCMV infection

**Fig 4:**
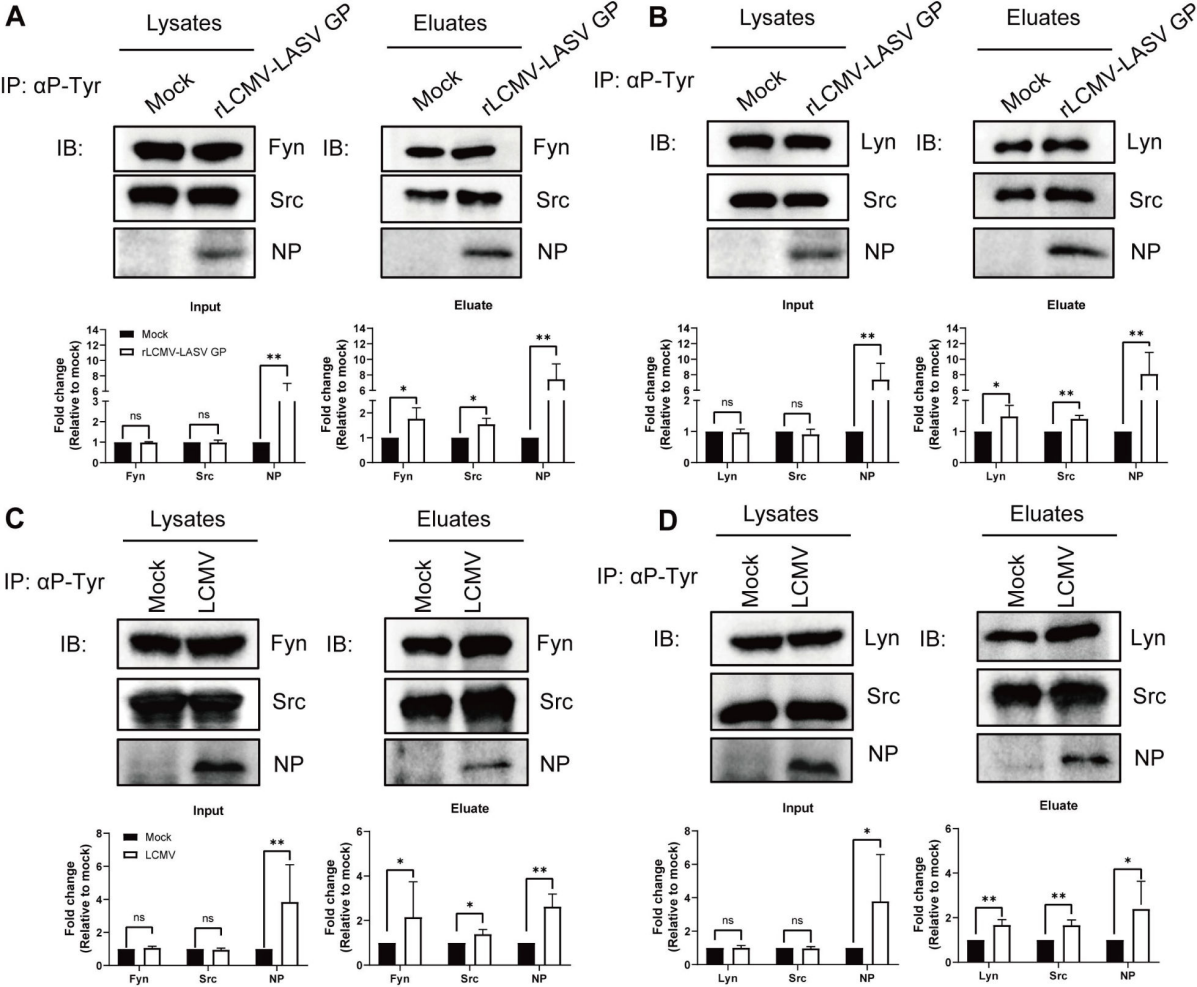
Analysis of Fyn and Lyn phosphorylation by IP following viral infection. A549 cells were transfected with pcDNA3.1-Fyn and pcDNA3.1-Lyn. The cells starved of serum for 24 h were infected with rLCMV-LASV GP or LCMV (MOI = 1). The cells were harvested 15 min after infection. Activation of Fyn, Lyn, and Src upon rLCMV-LASV GP (**A, B**) or LCMV (**C, D**) infection was measured by immunoprecipitation first on anti-phospho-tyrosine antibodies, immunoblotting with specific SFK antibodies, and quantitated using ImageJ software. IP analysis of Fyn (**A**) and Lyn (**B**) phosphorylation on rLCMV-LASV GP-infection. IP analysis of Fyn (**C**) and Lyn (**D**) phosphorylation on LCMV infection. Data shown are expressed as mean±SD of three independent experiments. The statistical significance was calculated by *t*-test; ns, not significant; *P < 0.05; **P < 0.01.

To evaluate the phosphorylation of Fyn and Lyn kinases post-rLCMV-LASV GP infection, immunoprecipitation (IP) assays were carried out. As shown in Fig. 4A, the tyrosine phosphorylation of Fyn and Src family kinases was markedly elevated in rLCMV-LASV GP-infected cells compared to uninfected controls. This observation aligned with our findings shown in Fig. 2, which showed enhanced SFK phosphorylation in infected cells. Similar results in Lyn phosphorylation to those of Fyn were also observed between infected and mock groups (Fig. 4B). To further verify the phosphorylation after rLCMV-LASV GP infection, a reverse IP assay was performed. Cell lysates were incubated with protein A/G magnetic beads, followed by an overnight incubation with the Fyn-, Lyn-, and Src-antibodies. Protein phosphorylation was subsequently assessed using phosphotyrosine-specific immunoblotting. In accordance with the results, rLCMV-LASV GP infection significantly increased tyrosine phosphorylation of Fyn, Lyn, and Src (Fig. S1).

Furthermore, to validate the roles of Fyn and Lyn in arenaviral pathogenesis further, we extended our analysis to LCMV, a prototypic pathogen of the arenavirus. As expected, the upregulation of Fyn, Lyn, and Src family kinase phosphorylation was observed by Western blot in cell samples collected after LCMV infection, which was consistent with the results of rLCMV-LASV GP infection (Fig. 4C and D).

### Knocking down the Fyn and Lyn kinases inhibited the infection of LCMV

**Fig 5:**
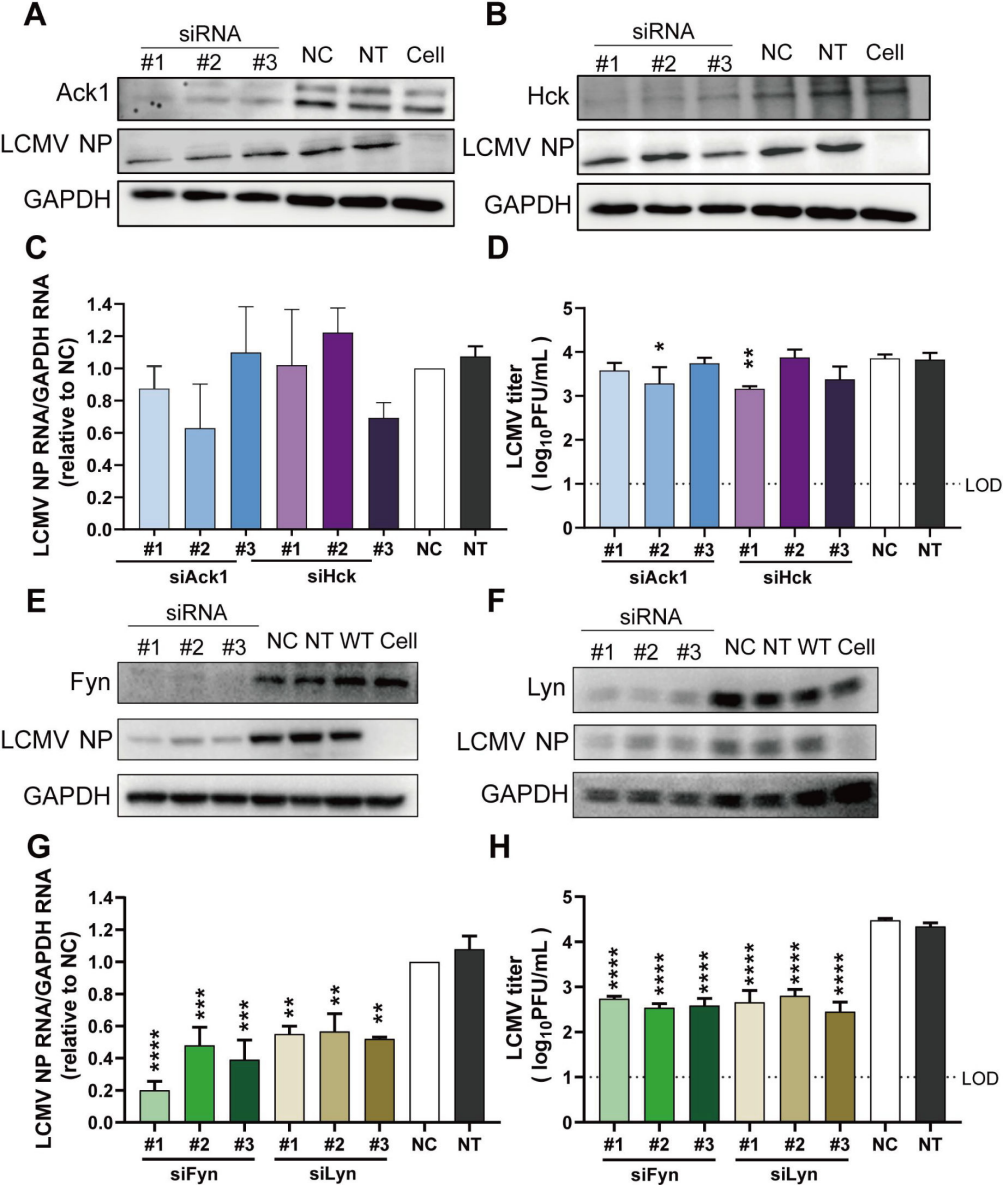
The effect of Ack1/Hck or Fyn/Lyn knockdown on LCMV infection. Following the same procedure as described before, (**A, B**) immunoblot analysis of viral protein expression with Ack1/Hck knockdown, infected with LCMV. (**C**) qRT-PCR quantification of viral RNA. (**D**) Plaque assay for infectious viral titers. (**E, F**) Immunoblot profiling of viral protein levels with Fyn/Lyn knockdown in LCMV-infected cells. (**G**) qRT-PCR quantification of viral mRNA expression. (**H**) Viral plaque assay for LCMV production across Fyn/Lyn knockdown. The significant differences were analyzed compared with the NC group. NC, negative control; NT, DEPC-treatment (DEPC-treated water) control; WT, viral infection group; cell, non-infection group; LOD, limit of detection. Data shown are expressed as mean±SD of three independent experiments. The statistical significance was calculated by one-way ANOVA; *P < 0.05, **P < 0.01, ***P < 0.001, and ****P < 0.0001.

To investigate whether the roles of Ack1, Hck, Fyn, and Lyn during LCMV infection differed from those during rLCMV-LASV GP infection, knockdown of Ack1/Hck and Fyn/Lyn experiments was performed in A549 cells. The effect on LCMV infection was analyzed by measuring the viral NP expression levels, genomic RNA levels, and viral titers. The results showed that knockdown of Ack1 and Hck did not cause significant alterations in the expression levels of LCMV NP (Fig. 5A through C). One out of three siRNAs targeting each kinase yielded a significant reduction in viral titers released into the supernatant, with decreases of 0.6 log and 0.7 log, respectively (Fig. 5D), indicating that Ack1 and Hck kinases are not essential for LCMV infection.

In contrast, transfection with multiple pairs of siRNAs targeting Fyn and Lyn resulted in a marked inhibition of viral infection. Consistent with rLCMV-LASV GP infection, Fyn (Fig. 5E) and Lyn (Fig. 5F) knockdown markedly reduced the expression levels of viral NP protein, suppressed viral mRNA accumulation, and diminished the supernatant viral titer (Fig. 5G and H). These data demonstrated that the knockdown of Fyn and Lyn kinases significantly reduces virus replication, establishing their essential roles in both rLCMV-LASV GP and LCMV infection.

### The inhibitors of Src kinases and downstream STAT3 suppress the infection of LCMV

To verify the essential role of these kinases, which are known to serve as promising host-directed antiviral targets, saracatinib, a potent inhibitor of SFKs, was selected to evaluate the antiviral effect (20). It was verified that saracatinib still exerted an inhibitory effect on the activity of SFKs in infected A549 cells (Fig. S3). Also, it has been previously reported that STAT3 activation is associated with arenavirus pathogenesis (21). Furthermore, C188-9, an additional inhibitor that targets the downstream molecule STAT3, was employed to confirm the antiviral potential of these kinases.

**Fig 6:**
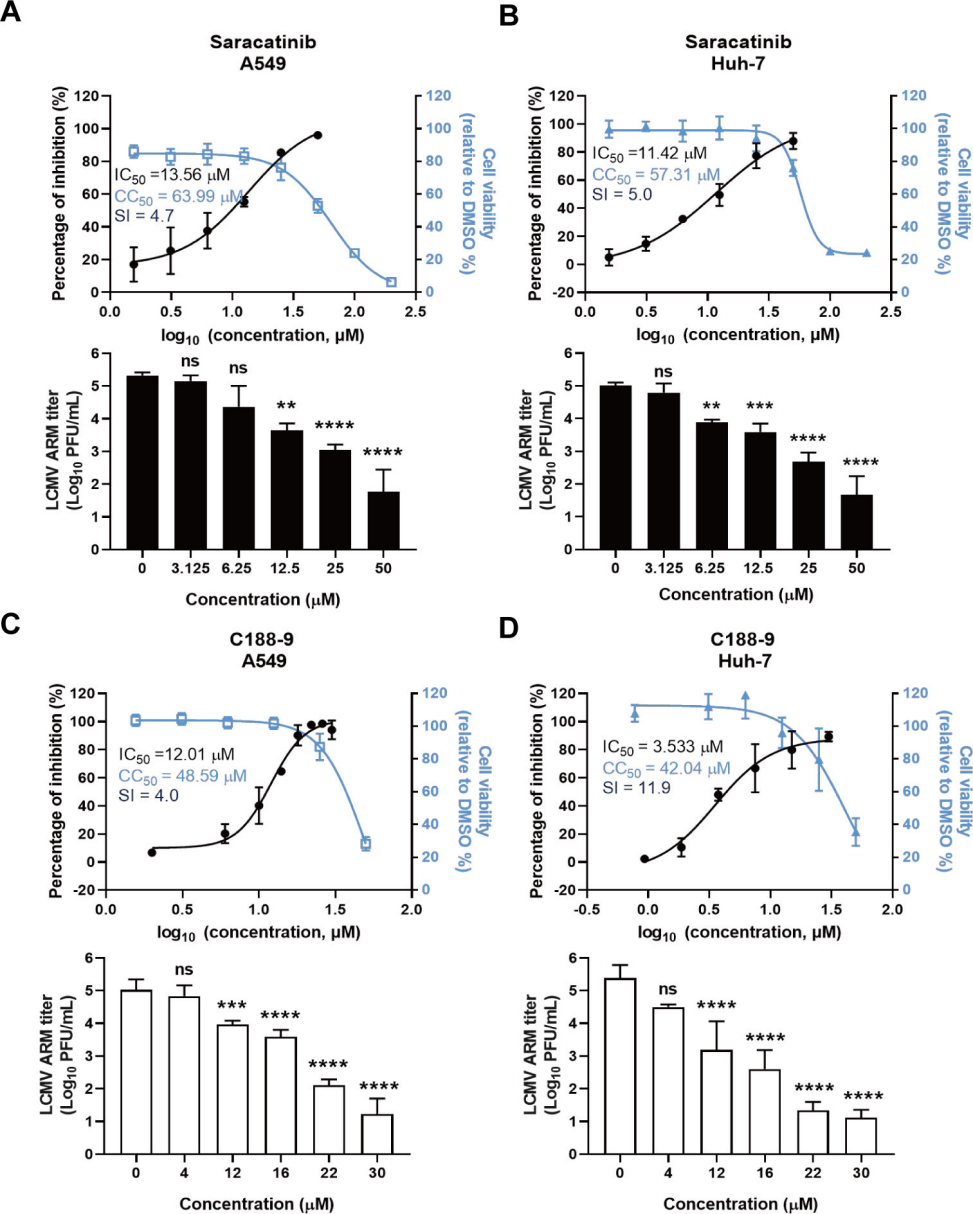
Inhibitory effects of saracatinib and C188-9 on LCMV Arm infection in A549 cells and Huh-7 cells. (**A, B**) Measurement of the effects of saracatinib on LCMV Arm in A549 (**A**) and Huh-7 (**B**) cells by qRT-PCR and plaque assay. (**C, D**) Measurement of the effects of C188-9 on LCMV Arm in A549 (**C**) and Huh-7 (**D**) cells by qRT-PCR and plaque assay. qRT-PCR was used to quantify viral RNA (left axis) and cytotoxicity by CCK-8 assay (right axis) in A549 cells (left) and Huh-7 cells (right). These CC_50_ values were consistent with the previous data. SI was calculated as SI = CC_50_/IC_50_. Cells were infected with LCMV Arm (MOI = 0.1) for 1 h in the presence of various drug concentrations and incubated with the drug for 24 h. Data shown are expressed as mean±SD of three independent experiments. Significance was determined by one-way ANOVA; ns, not significant; **P < 0.01, ***P < 0.001, and ****P < 0.0001.

The inhibitory effects of saracatinib and C188-9 on LCMV strains were evaluated in A549 and Huh-7 cells using immunofluorescence assay (IFA) and qRT-PCR and immune plaque titer assays. In A549 cells, qRT-PCR revealed dose-dependent inhibition of LCMV Armstrong strain (LCMV Arm) by saracatinib, with C188-9 exhibiting slightly superior efficacy. The IC_50_ of saracatinib against LCMV Arm was 13.56 μM, and the IC_50_ of C188-9 against LCMV Arm was 12.76 μM. The titers decreased by 3.5 log values and 3.7 log values, respectively, at the highest concentration tested (Fig. 6A and B). Both inhibitors displayed comparable cytotoxicity. In Huh-7 cells, saracatinib and C188-9 also displayed dose-dependent inhibition of LCMV Arm infection, with effective concentration ranges consistent with those in A549 cells. The IC_50_ of saracatinib against LCMV Arm was 11.42 μM, and the IC_50_ of C188-9 was 3.533 μM. The titer was reduced by 3.3 and 4.2 log values, respectively, following treatment with the highest concentration tested (Fig. 6C and D). Collectively, saracatinib and C188-9 inhibited LCMV Arm infection in a dose-dependent manner across various human cell lines.

**Fig 7:**
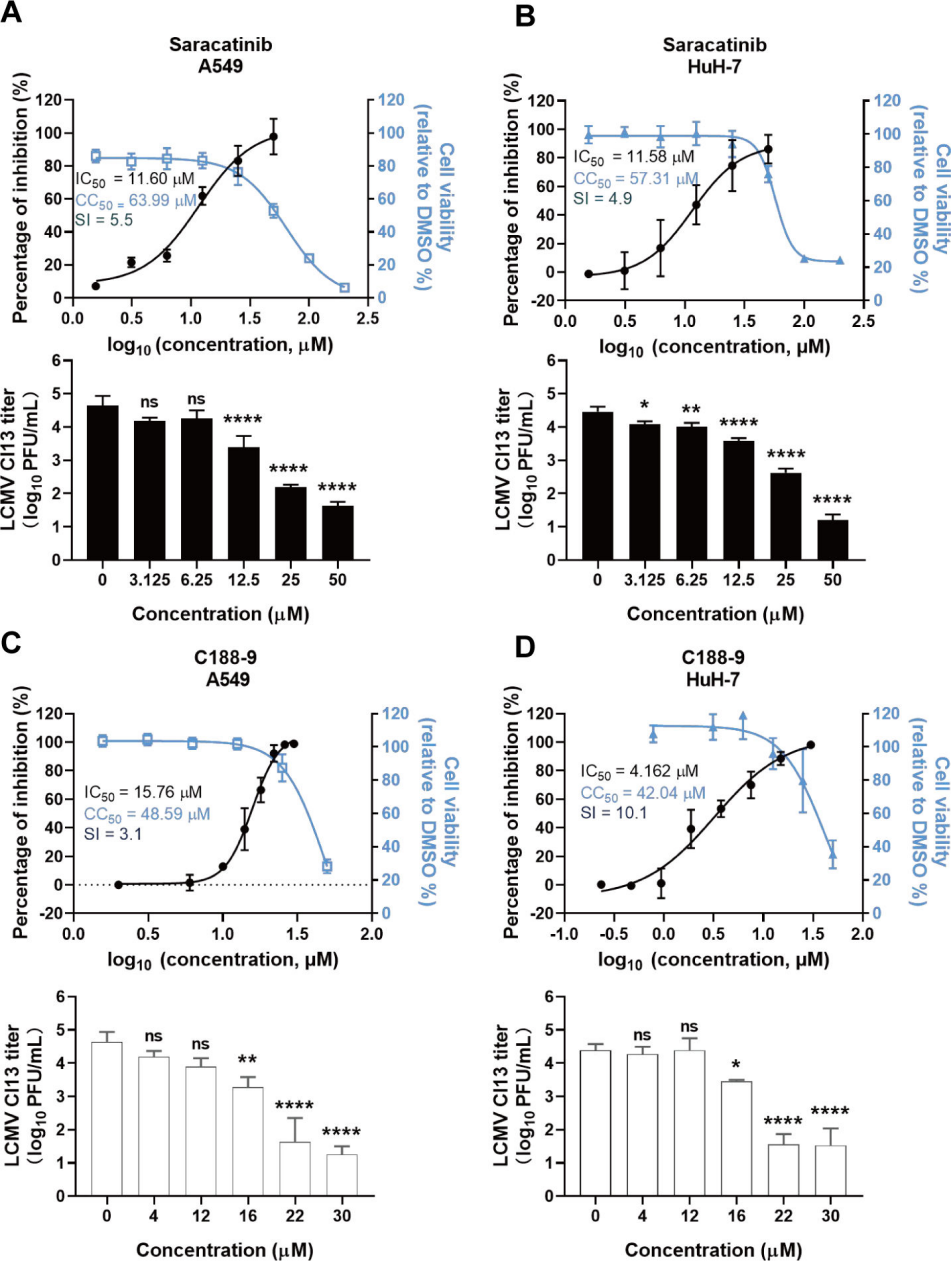
Inhibitory effects of saracatinib and C188-9 on LCMV Cl13 infection in A549 cells and Huh-7 cells. (**A, B**) Measurement of the effects of saracatinib on LCMV Cl13 in A549 (**A**) and Huh-7 (**B**) cells by qRT-PCR and plaque assay. (**C, D**) Measurement of the effects of C188-9 on LCMV Cl13 in A549 (**C**) and Huh-7 (**D**) cells by qRT-PCR and plaque assay. qRT-PCR was used to quantify viral RNA (left axis) and cytotoxicity by CCK-8 assay (right axis) in A549 cells (left) and Huh-7 cells (right). These CC_50_ values were consistent with the previous data. SI was calculated as SI = CC_50_/IC_50_. Cells were infected with LCMV Cl13 (MOI = 0.1) for 1 h in the presence of various drug concentrations and incubated with the drug for 24 h. Data shown are expressed as mean±SD of three independent experiments. Significance was determined by one-way ANOVA; ns, not significant; *P < 0.05, **P < 0.01, and ****P < 0.0001.

To evaluate the efficacy of these compounds against LCMV, we assessed the activity of saracatinib and C188-9 against the LCMV Clone 13 strain (LCMV Cl13), a distinct LCMV strain, using the aforementioned methods. In A549 cells, the IC_50_ of saracatinib against LCMV Cl13 was 11.60 μM (Fig. 7A), while that of C188-9 was 15.76 μM (Fig. 7C). Plaque assays of culture supernatants demonstrated that saracatinib reduced viral titers by 2 orders of magnitude compared with dimethyl sulfoxide (DMSO) controls in a range of 25–50 μM, indicating significant inhibition by saracatinib of LCMV Cl13 production (Fig. 7A and B). Similarly, C188-9 treatment also significantly inhibited the production of LCMV Cl13 progeny virus (Fig. 7C and D). In summary, saracatinib and C188-9 dose-dependently inhibited LCMV Cl13 infection, suppressing viral proliferation in cells and the production of progeny virus.

Thus, the results indicated that saracatinib and C188-9, the inhibitors of Src family kinases and transcriptional regulator STAT3, exhibited potent inhibitory effects on both LCMV Arm and LCMV Cl13 infections. These compounds dose-dependently inhibit viral protein and mRNA synthesis, suppressing progeny virus production within a noncytotoxic concentration range.

### The Src kinase inhibitor saracatinib exhibits antiviral efficacy on LCMV *in vivo*

**Fig 8:**
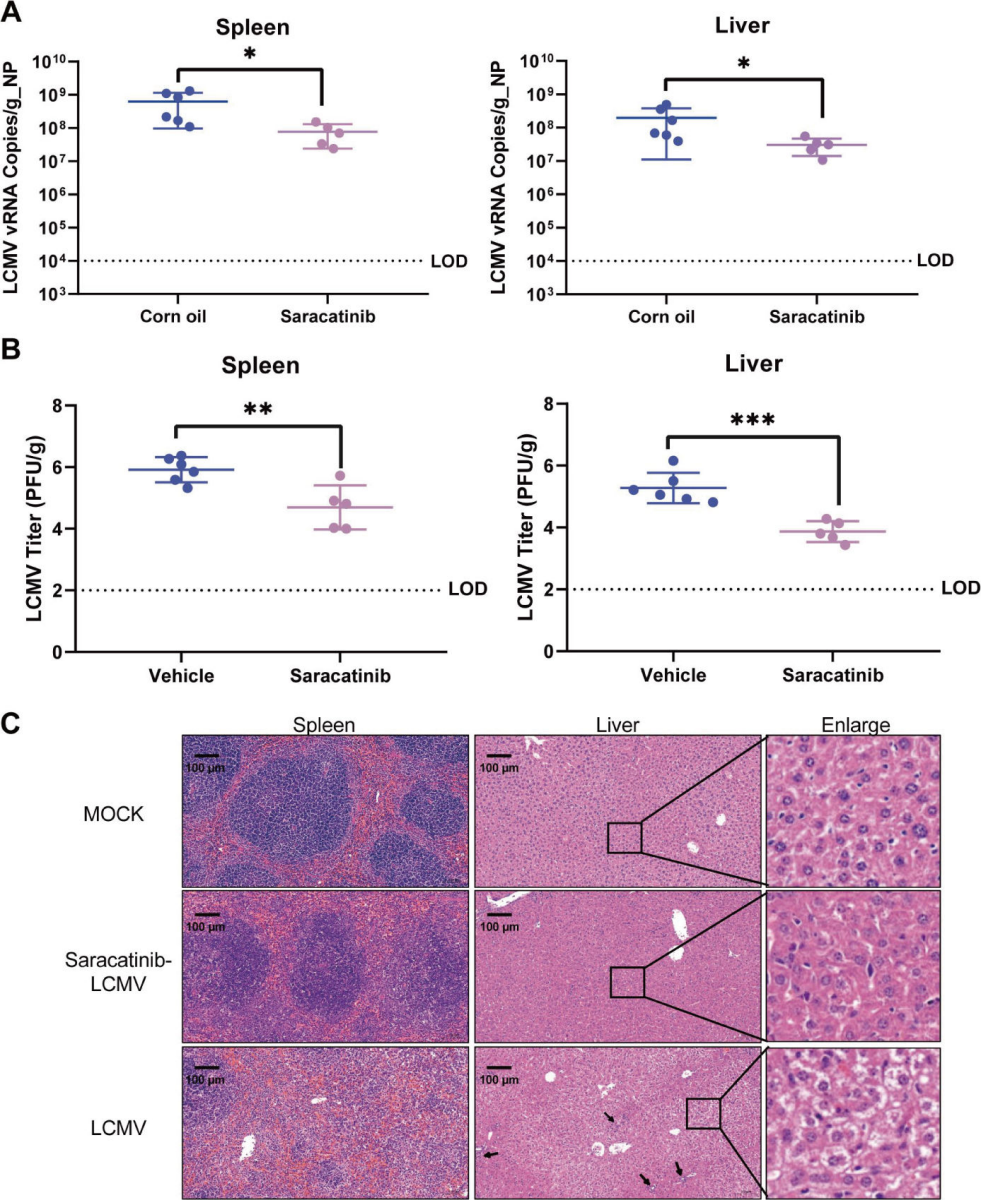
Effects of saracatinib on LCMV infection in C57BL/6JSmoc‐*Prf1^em1Smoc^* mice. (**A**) qRT-PCR and (**B**) titers of spleen and liver tissues dissociated at 5 dpi. (**C**) Spleen and liver tissues collected at 5 dpi were stained with HE to observe pathological damage (black arrow, inflammatory cell infiltration; enlargement, cellular edema). Image scale bar, 100 μm. Significance in panels A and B was determined by *t*-test; *P < 0.05, **P < 0.01, and ***P < 0.001.

To assess the *in vivo* antiviral potential of saracatinib, we employed C57BL/6JSmoc‐*Prf1^em1Smoc^* mice—a well-established preclinical model for LCMV studies—due to their heightened viral susceptibility. Given peak viral loads in splenic and hepatic tissues at 5 days post-infection (dpi), organs were collected at this critical time point for viral load quantification. Saracatinib significantly lowered viral genomic levels (Fig. 8A) and LCMV titers (Fig. 8B) in both spleen and liver compared to vehicle controls, corroborating the therapeutic capacity of saracatinib.

Histopathological evaluation further validated the antiviral efficacy of saracatinib. LCMV-infected controls exhibited pronounced hepatic damage, including lymphocytic infiltration and parenchymal edema, along with splenic white pulp atrophy. Saracatinib treatment ameliorated LCMV-induced tissue damage, preserving hepatic architecture and splenic follicular integrity (Fig. 8C). These findings collectively establish saracatinib as a potent inhibitor of LCMV replication *in vivo*, effectively attenuating viral burden and mitigating infection-associated histopathology.

### The Src kinase inhibitor saracatinib suppresses the infection of the authentic LASV

**Fig 9:**
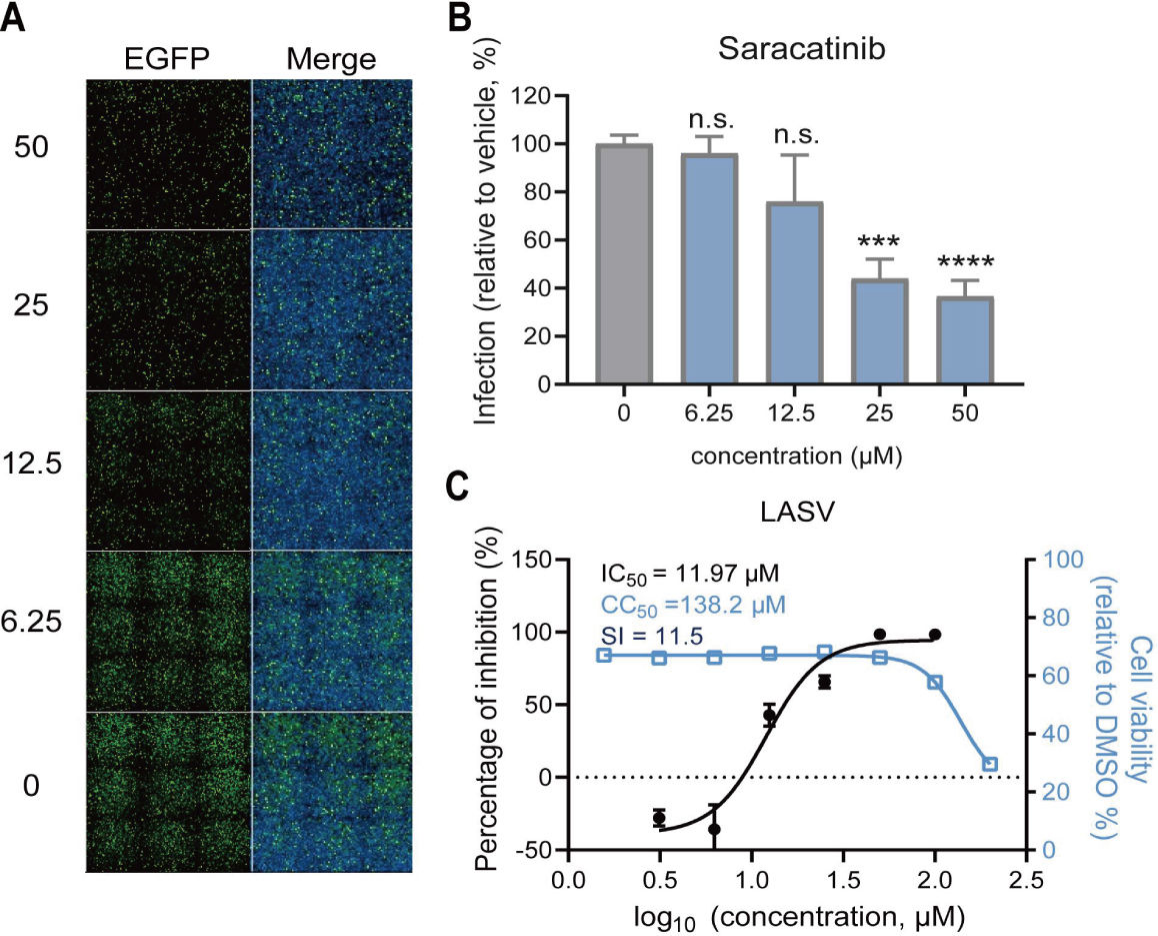
Inhibitory effects of saracatinib on authentic LASV infection. (**A**) IFA and (**B**) quantification of the saracatinib inhibitory rate of LASV infection on Vero E6 cells. Vero E6 cells were pre-incubated with saracatinib or vehicle at 37°C for 1 h, followed by incubation with LASV (MOI, 0.1) at 4°C for 1 h. Cells were stained with an anti-LASV NP antibody (green), and the nuclei were stained with DAPI (blue). (**C**) Measurement of the effects of saracatinib on LASV in Vero E6 cells by qRT-PCR. Data shown are expressed as mean±SD of three independent experiments. The statistical significance was calculated by one-way ANOVA. ns, not significant; ***P < 0.001; ****P < 0.0001.

To determine whether the Src kinase inhibitor saracatinib has an antiviral effect on the authentic LASV, we evaluated the inhibitory efficacy of saracatinib against LASV infection *in vitro* by using IFA and qRT-PCR assay. Vero E6 cells were infected with LASV and treated with serially diluted concentrations of saracatinib, with vehicle-treated controls in parallel. At 48 h post-infection (hpi), quantitative analysis of IFA-positive cells revealed a dose-dependent suppression of viral infectivity (Fig. 9A and B). The IC_50_ (11.97 μM) was calculated based on the analysis of qRT-PCR (Fig. 9C), which is similar to the IC_50_ result of saracatinib against LCMV.

## DISCUSSION

In this study, we demonstrated that both rLCMV-LASV GP and LCMV induce the phosphorylation of Fyn and Lyn during cellular infection, confirming the involvement of these SFKs in arenavirus infection. We further identified that Fyn and Lyn, two key members of the SFKs, are critical in viral infection. Fyn is known to regulate intracellular signal transduction and contribute to core cellular processes, including cell migration, adhesion, growth, and differentiation (22, 23). During infection with group B coxsackieviruses (CVB), Fyn was activated upon the binding of CVB to the cell surface decay-accelerating factor, mediating CVB entry into apical cells via caveolin-associated vesicles (24). Lyn modulates two distinct physiological pathways: it regulates integrin signaling and orchestrates adaptive immune responses (25–28). In human T-cell leukemia virus type 1 (HTLV-1) infection, Lyn interacted with the interleukin-2 beta receptor (IL-2Rβ) and Janus kinase 3 (JAK3), thereby promoting the phosphorylation of signal transducer and activator of transcription 5 (STAT5) protein (19, 29). Additionally, previous studies have shown that flaviviruses utilize a Lyn-dependent released pathway in LC3+ secretory organelles to evade circulating antibodies and affect tissue tropism (30). Collectively, these findings suggest that both Fyn/Lyn kinases play important roles in multiple stages of viral infection. Our results revealed that Fyn/Lyn kinases were activated during the early phase of arenavirus infection, a process that may be associated with viral entry. Moreover, knockdown of these two genes resulted in the inhibition of both viral genomic RNA replication and viral particle production. Notably, although Fyn/Lyn knockdown exerted similar effects on NP expression for LCMV (Fig. 5E and F) and rLCMV-LASV GP (Fig. 3E and F)—consistent with Fyn/Lyn regulating early replication steps (e.g., viral entry and initial NP synthesis) common to both viruses—the inhibitory effect on LCMV titer (Fig. 5D) was stronger than that on rLCMV-LASV GP (Fig. 3H). This discrepancy may suggest that Fyn and Lyn kinases are involved in distinct mechanisms underlying infection by the two viruses. The key difference between LCMV and rLCMV-LASV GP resides in their glycoprotein (GP). In arenaviruses, GP was not only involved in viral entry but also contributed to the release of progeny viruses (31). Knockdown of Fyn/Lyn significantly impaired the viral titer of LCMV released into the supernatant. This observation implied that Fyn and Lyn kinases were also involved in other stages of arenavirus infection and that their functional roles might not be limited to the viral entry process. Further studies are required to clarify the specific mechanisms by which they participate in these infection processes.

Drug repurposing represents a critical strategy in drug development and serves as an effective approach to control the spread of highly pathogenic pathogens such as Lassa fever. Saracatinib, a Src family kinase inhibitor approved by the FDA in 2019 for the treatment of idiopathic pulmonary fibrosis (32), has previously been proven to exert antiviral effects against viruses including Middle East respiratory syndrome coronavirus (MERS-CoV) and herpes simplex virus type 1 (HSV-1) (33–36). Notably, saracatinib inhibits viral infection by targeting common signaling pathways and molecular mechanisms, suggesting its potential as a broad-spectrum antiviral agent. Our study focused on investigating the antiviral effects of the kinase inhibitor saracatinib against arenavirus infection *in vitro* and *in vivo*. The results showed that saracatinib had dose-dependent inhibitory effects on different LCMV strains and LASV infection. This study expanded the potential application scope of saracatinib and provided a theoretical basis for its repurposing as an anti-arenaviral agent.

In summary, our results regarding the activation of Fyn and Lyn kinases during LASV and LCMV entry provide new insights into the interaction mechanisms between arenaviruses and host cells. The mechanisms characterized in this study deepen our understanding of the pathological features and pathogenic mechanisms of arenaviruses and further identify novel targets for the prevention and treatment of arenaviral infections.

## MATERIALS AND METHODS

### Cell lines and viruses

Baby hamster kidney (BHK-21) cells, human embryonic kidney (HEK) 293T cells, human liver epithelial-like cells (Huh-7) cells, T7 RNA polymerase-stably expressing (BSR-T7) cells, and chlorocebus aethiops kidney (Vero E6) cells were cultured in Dulbecco’s modified Eagle’s medium (DMEM; HyClone) supplemented with 10% fetal bovine serum (FBS; Gibco). Human alveolar basal epithelial (A549) cells were cultured in Dulbecco’s modified Eagle medium/Nutrient Mixture F-12 (DMEM/F-12; HyClone) with 10% FBS. Cells were cultured at 37°C with 5% CO_2_.

LCMV Armstrong was rescued as described previously. Briefly, a mixture of plasmids containing LCMV-S (Armstrong, GenBank: AY847350.1) and LCMV-L (Armstrong, GenBank: AY847351.1) was transfected into T7 RNA polymerase expressing BSR-T7 cells. The supernatant was collected 72 h later and inoculated into BHK-21 cells for rLCMV amplification. LCMV Cl13 was rescued consistent with LCMV Armstrong, and three mutations were introduced in L and S segments, including K1079Q on the L segment and F260L and N176D on the S segment. Recombinant LCMV expressing LASV glycoprotein (rLCMV-LASV GP) was generated as described previously. Briefly, rLCMV-LASV GP was generated by replacing the native LCMV GPC with the LASV GPC in the LCMV S segment; the modified S segments were co-transfected along with the LCMV L segments into BSR-T7 cells. The initially rescued viruses were transferred into BHK-21 cells for rLCMV-LASV GP amplification. The authentic LASV strain Lassa_HX strain (Science Data Bank, CSTR: 31253.11.sciencedb.22517) was maintained at the Wuhan Institute of Virology, Chinese Academy of Sciences.

### Screening of activated receptor tyrosine kinases in rLCMV-LASV GP-infected cell extracts

After serum starvation for 24 h, the 293T and A549 cells were infected with rLCMV-LASV GP at an MOI of 10. The sample mixtures were incubated for 1 h on ice and incubated for 15 min at 37°C for infection. Cells were then harvested and lysed. The subsequent procedures were performed according to Abcam’s human RTK phosphorylation antibody array membrane instructions (ab193662; Abcam, Cambridge, UK) to measure the phosphorylation of 71 kinases in cell lysates. The membrane signals were acquired by chemiluminescence detection. The levels of kinase phosphorylation were quantified using ImageJ software.

### Immunoblotting assay for kinase phosphorylation

After A549 cells were serum-starved for 24 h, they were placed in an ice bath at 4°C for 30 min to reach temperature equilibration. Then, the cells were co-incubated with rLCMV-LASV GP at an MOI of 0.1 and 1 on ice for 1 h to allow virus attachment. A mock group (treated with virus-free DMEM under the same temperature conditions) was set up in parallel to exclude experimental errors caused by operational or temperature variations. Following the 4°C co-incubation, cells were transferred to a 37°C incubator to initiate viral entry—this transfer moment was defined as “0 min post-infection.” At 0, 5, 10, 15, 30, and 45 min post-transfer, cell supernatants were collected, and cells were lysed with RIPA buffer (Beyotime, Shanghai, China). Proteins were separated by sodium dodecyl sulfate (SDS) polyacrylamide gel electrophoresis and then transferred to a polyvinylidene fluoride membrane. The membrane was stained with the primary antibodies Phospho-Src Family Tyr416 (Cell Signaling Technology; dilution 1:1,000) or Src Rabbit mAb (Cell Signaling Technology; dilution 1:1,000). Densitometry analysis was performed using ImageJ over three independent experiments.

### siRNA-mediated gene knockdown

Stock solutions (50 nM per well in 24-well plates) of siRNA listed in Table S1 (Gene Pharma) and siRNA control (named NC, UUC UCC GAA CGU GUC ACG UTT) were prepared using RNase-free water. siRNA transfection was performed with RNAiMAX (Invitrogen) according to instructions; the siRNA used in per well was at a final concentration of 5 pmol. The master mix of RNAiMAX reagent and Opti-MEM was incubated with siRNA for 15 min at room temperature, after which cells were incubated with the transfection mix for 24 h at 37°C. Time-course growth kinetic assays were performed in knockdown cells using the optimal siRNAs, demonstrating that the knockdown efficiency was stably maintained from 24 h to 48 h (Fig. S4). We chose to incubate cells with the transfection mixture at 37°C for 24 h. Subsequently, the medium was removed and replaced with fresh DMEM supplemented with 2% FBS. Cells were infected with rLCMV-LASV GP and LCMV at an MOI of 0.1 for 24 h.

### Immunoprecipitation assays

A549 cells were counted and adjusted the concentration to 2.5 × 10^6^ cells with complete medium to the 10 cm dish. Cells were transfected with pcDNA3.1-Fyn and pcDNA3.1-Lyn. After 24 h of serum starvation post-transfection, the cells were infected with rLCMV-LASV GP or LCMV Cl13 at an MOI of 1. At 15 min post-infection, the cells were harvested, and the proteins were extracted with Pierce IP Lysis Buffer (Thermo Fisher Scientific). A portion of the cell lysate was used as lysate samples. The other portion was used as eluate samples, which were incubated with Phospho-Tyrosine Mouse mAb P-Tyr-100 Magnetic Bead Conjugate (Cell Signaling Technology; dilution 1:20) at 4°C overnight. The lysate and antibody (immune complex) solution was transferred to a test tube containing the pellet of pre-washed magnetic beads, and the mixture was incubated with rotation at room temperature for 20 min. Then, the magnetic beads were pelleted using a magnetic separation rack. Subsequently, the beads were washed with PBST, and the eluates were collected with elution buffer. The lysates and eluates were analyzed by Western blotting. The primary antibodies were as follows: anti-Fyn mAb (Cell Signaling Technology; 1:1,000), anti-Lyn mAb (Cell Signaling Technology; 1:1,000) and anti-Src family kinases (Cell Signaling Technology; 1:1,000), and anti-LCMV NP pAb (prepared in this laboratory, 1:1,000). The secondary antibodies were as follows: HRP goat anti-mouse IgG and HRP goat anti-rabbit IgG (both from ABclonal, 1:5,000).

### Western blotting assay

Samples containing equal amounts of protein were separated by SDS-PAGE and transferred to polyvinylidene difluoride (PVDF) membranes (Millipore, Burlington, MA, USA). The membrane was blocked with 3% bovine serum albumin (BSA) in TBST for 2 h and then incubated overnight at 4°C with the primary antibody, followed by incubation with secondary antibodies at room temperature for 45 min. The membranes were immersed in an enhanced chemiluminescence solution (Millipore), and protein bands were visualized using enhanced chemiluminescence (ECL) and a ChemiDoc imager (Bio-Rad Laboratories).

### Antiviral activity assay and drug cytotoxicity assay

For the antiviral assay, A549 and Huh-7 cells were prepared by growing to a density of 2 × 10^5^ cells/mL, and the cells were added into 24-well plates and grown at 37°C with 5% CO_2_ for 16 h. The cells were pretreated with the gradient-diluted inhibitors for 1 h and infected with LCMV Armstrong or LCMV Cl13 at an MOI of 0.1 for 1 h in the absence or in the presence of drugs. After incubation, the supernatant was removed and replaced with fresh drug-containing medium and further maintained until 24 h post-infection. The antiviral effects of inhibitors were measured by qRT-PCR and immune plaque assay.

For drug cytotoxicity assays, cells were seeded into 96-well plates at a density of 2 × 10^5^ cells/mL. Appropriate concentrations of the compounds were added to the medium 16 h later. After incubating for 24 h, the relative number of surviving cells was measured using the Cell Counting Kit 8 assay (Beyotime), followed by the manufacturer’s instructions. The absorbance was measured at a 450 nm wavelength. The CC_50_ values were calculated using GraphPad Prism 8.0 software.

### Immunofluorescence staining assay

All LASV infections were performed in biosafety level 4 (BSL-4) laboratory of the National Biosafety Laboratory (Wuhan), Chinese Academy of Sciences. Vero-E6 cells were seeded at a density of 1 × 10^4^ cells per well in 96-well plates and incubated at 37°C. The cells were pretreated with the gradient-diluted inhibitors for 1 h and infected with LASV at an MOI of 0.1 for 1 h in the absence or in the presence of drugs. After incubation, the supernatant was removed and replaced with fresh drug-containing medium and further maintained until 48 h post-infection.

LASV-infected Vero-E6 cells were fixed in 4% paraformaldehyde after the culture medium was removed. The cells were then washed three times with PBS and permeabilized by incubation with 0.02% Triton X-100 for 20 min. Subsequently, the cells were blocked with 5% BSA at 37°C for 2 h, followed by using a primary antibody against LASV-NP (diluted 1:50). The samples were washed, and a secondary antibody (CoraLite488-conjugated goat anti-rabbit IgG, Proteintech, 1:500) was used. Cell nuclei were stained using 4′,6-diamidino-2-phenylindole (DAPI) (Solarbio, 1 mg/mL).

Fluorescence images were captured using an Operetta high-content imaging system (PerkinElmer), and the percentages of infected and DAPI-positive cells were calculated using the associated Harmony 3.5 software.

### Quantitative real-time PCR assay

Total RNA was extracted from LCMV-infected cells by using TRIzol reagent (Invitrogen). RNA from tissue samples was extracted with the virus DNA/RNA extraction kit 2.0 (Vazyme). Reverse transcription was performed with HiScript III Q RT SuperMix (Vazyme). The following qRT-PCR was performed with the specific primers listed in Table S2. mRNA levels of LCMV NP and LASV NP gene transcripts were normalized to the transcript level of the housekeeping GAPDH gene. Relative gene expression levels were calculated using the 2^−∆∆Ct^ method. GAPDH was used as an internal control, and the threshold cycle (∆Ct) value was calculated as ∆Ct = Ct (NP) – Ct (GAPDH).

### Immune plaque assay

BHK-21 cells were seeded at a density of 2 × 10^4^ cells per well in 24-well plates and incubated at 37°C for 16 h. Cells were inoculated with infective supernatants in a 10-fold serial dilution using serum-free DMEM and incubated at 37°C for 1 h, while the untreated cells were regarded as a negative control. After removal of virus suspension, cells were overlaid with DMEM (containing 2% FBS) containing 1% methyl cellulose (Solarbio) at 37°C for 72 h.

Infected cells were fixed in 4% paraformaldehyde and permeabilized with 0.1% Triton-X-100 for 30 min at room temperature. The cells were again washed thrice with PBS and blocked with 1% BSA for 2 h. The antibodies used were as follows: primary antibody anti-LCMV NP protein (diluted 1:10, M104; GeneTex) and secondary antibody goat anti-mouse HRP-conjugated IgG (diluted 1:500; ABclonal). This was followed by staining of the viral titer using an Enhanced HRP-DAB Chromogenic Kit (TIANGEN) according to the manufacturer’s instructions. Virus titer was determined by counting the number of viral plaques.

### LCMV challenge on animal models

Saracatinib (HY-10234) and C188-9 (HY-112288), employed in this research, were procured from MedChemExpress and were solubilized in DMSO.

C57BL/6JSmoc-*Prf1^em1Smoc^* mice (6–8 weeks old) were randomly assigned to three groups: the non-infected group (mock), the vehicle treated (corn oil) group, and saracatinib therapeutic group. For infection, mice were infected intraperitoneally (i.p.) with a dose of 2 × 10^5^ PFU LCMV Arm. Following infection, saracatinib (30 mg/kg body weight per mouse) or vehicle was orally administered (p.o.) to mice every day from 0 to 5 dpi. Body weights and symptoms were monitored every day. Mice were sacrificed at 5 dpi for virological and histopathological analyses. Spleen and liver samples were collected for immune plaque assay, qRT-PCR, and histopathology observation. Briefly, tissues were dissected and ground with 1 mL DMEM, and the supernatants containing virus particles after centrifugation were prepared to determine the viral titer using an immune plaque assay and LCMV mRNA levels using qRT-PCR. The viral copies per gram were calculated on a standard curve produced using serial tenfold dilutions of LCMV NP plasmid. Hematoxylin-eosin (HE) staining (Servicebio) was performed on tissue sections to evaluate pathological changes.

## Data Availability

All data supporting the findings of this study are available within the paper and its supplemental material.

## SUPPLEMENTAL MATERIAL

Supplemental material jvi.00241-26-s0001.docx Supplemental methods, Fig. S1 to S4, and Tables S1 and S2.

ASM does not own the copyrights to Supplemental Material that may be linked to, or accessed through, an article. The authors have granted ASM a non-exclusive, world-wide license to publish the Supplemental Material files. Please contact the corresponding author directly for reuse.

## References

[1] Klitting R, Mehta SB, Oguzie JU, Oluniyi PE, Pauthner MG, Siddle KJ, Andersen KG, Happi CT, Sabeti PC. 2023. Lassa virus genetics. Curr Top Microbiol Immunol 440:23–65. doi:10.1007/82_2020_212 32418034

[2] Peng R, Xu X, Jing J, Wang M, Peng Q, Liu S, Wu Y, Bao X, Wang P, Qi J, Gao GF, Shi Y. 2020. Structural insight into arenavirus replication machinery. Nature 579:615–619. doi:10.1038/s41586-020-2114-2 32214249

[3] Arefin A, Ismail Ema T, Islam T, Hossen S, Islam T, Al Azad S, Uddin Badal N, Islam A, Biswas P, Alam NU, Islam E, Anjum M, Masud A, Kamran S, Rahman A, Kumar Paul P. 2021. Target specificity of selective bioactive compounds in blocking α-dystroglycan receptor to suppress Lassa virus infection: an in silico approach. J Biomed Res 35:459–473. doi:10.7555/JBR.35.20210111 34857680 PMC8637655

[4] Goncalves AR, Moraz ML, Pasquato A, Helenius A, Lozach PY, Kunz S. 2013. Role of DC-SIGN in Lassa virus entry into human dendritic cells. J Virol 87:11504–11515. doi:10.1128/JVI.01893-13 23966408 PMC3807329

[5] Shimojima M, Ströher U, Ebihara H, Feldmann H, Kawaoka Y. 2012. Identification of cell surface molecules involved in dystroglycan-independent Lassa virus cell entry. J Virol 86:2067–2078. doi:10.1128/JVI.06451-11 22156524 PMC3302412

[6] Soares MM, King SW, Thorpe PE. 2008. Targeting inside-out phosphatidylserine as a therapeutic strategy for viral diseases. Nat Med 14:1357–1362. doi:10.1038/nm.1885 19029986 PMC2597367

[7] Brouillette RB, Phillips EK, Patel R, Mahauad-Fernandez W, Moller-Tank S, Rogers KJ, Dillard JA, Cooney AL, Martinez-Sobrido L, Okeoma C, Maury W. 2018. TIM-1 mediates dystroglycan-independent entry of Lassa virus. J Virol 92:e00093-18. doi:10.1128/JVI.00093-18 29875238 PMC6069209

[8] Zhou M, Wang S, Guo J, Liu Y, Cao J, Lan X, Jia X, Zhang B, Xiao G, Wang W. 2021. RNA interference screening reveals requirement for platelet-derived growth factor receptor beta in Japanese encephalitis virus infection. Antimicrob Agents Chemother 65:e00113-21. doi:10.1128/AAC.00113-21 33753340 PMC8316074

[9] Evans JP, Liu SL. 2020. Multifaceted roles of TIM-family proteins in virus-host interactions. Trends Microbiol 28:224–235. doi:10.1016/j.tim.2019.10.004 31732320 PMC7018592

[10] Meertens L, Labeau A, Dejarnac O, Cipriani S, Sinigaglia L, Bonnet-Madin L, Le Charpentier T, Hafirassou ML, Zamborlini A, Cao-Lormeau VM, Coulpier M, Missé D, Jouvenet N, Tabibiazar R, Gressens P, Schwartz O, Amara A. 2017. Axl mediates ZIKA virus entry in human glial cells and modulates innate immune responses. Cell Rep 18:324–333. doi:10.1016/j.celrep.2016.12.045 28076778

[11] Meertens L, Carnec X, Lecoin MP, Ramdasi R, Guivel-Benhassine F, Lew E, Lemke G, Schwartz O, Amara A. 2012. The TIM and TAM families of phosphatidylserine receptors mediate dengue virus entry. Cell Host Microbe 12:544–557. doi:10.1016/j.chom.2012.08.009 23084921 PMC3572209

[12] Eshaq AM, Flanagan TW, Hassan SY, Al Asheikh SA, Al-Amoudi WA, Santourlidis S, Hassan SL, Alamodi MO, Bendhack ML, Alamodi MO, Haikel Y, Megahed M, Hassan M. 2024. Non-receptor tyrosine kinases: their structure and mechanistic role in tumor progression and resistance. Cancers (Basel) 16:2754. doi:10.3390/cancers16152754 39123481 PMC11311543

[13] Boggon TJ, Eck MJ. 2004. Structure and regulation of Src family kinases. Oncogene 23:7918–7927. doi:10.1038/sj.onc.1208081 15489910

[14] Gocek E, Moulas AN, Studzinski GP. 2014. Non-receptor protein tyrosine kinases signaling pathways in normal and cancer cells. Crit Rev Clin Lab Sci 51:125–137. doi:10.3109/10408363.2013.874403 24446827

[15] Lian R, Dou X, Wang N, Li S, Xie J, Li X, Yang Y, Wen Y, Li H, Feng R. 2025. ICAM-1-mediated Src signaling pathway plays a pivotal role in encephalomyocarditis virus entry. J Virol 99:e0071525. doi:10.1128/jvi.00715-25 40631914 PMC12363205

[16] Fan TJ, Xie C, Li L, Jin X, Cui J, Wang JH. 2025. HIV-1 Nef activates proviral DNA transcription by recruiting Src kinase to phosphorylate host protein Nef-associated factor 1 to compromise its viral restrictive function. J Virol 99:e0028025. doi:10.1128/jvi.00280-25 40272155 PMC12090801

[17] Chen J, Fu Y, Li Y, Weng S, Wang H, He J, Dong C. 2025. Transferrin receptor 1 (TfR1) functions as an entry receptor for scale drop disease virus to invade the host cell via clathrin-mediated endocytosis. J Virol 99:e0067125. doi:10.1128/jvi.00671-25 40719463 PMC12363161

[18] Lingemann M, McCarty T, Liu X, Buchholz UJ, Surman S, Martin SE, Collins PL, Munir S. 2019. The alpha-1 subunit of the Na^+^,K^+^-ATPase (ATP1A1) is required for macropinocytic entry of respiratory syncytial virus (RSV) in human respiratory epithelial cells. PLoS Pathog 15:e1007963. doi:10.1371/journal.ppat.1007963 31381610 PMC6695199

[19] Gorbunova EE, Gavrilovskaya IN, Pepini T, Mackow ER. 2011. VEGFR2 and Src kinase inhibitors suppress Andes virus-induced endothelial cell permeability. J Virol 85:2296–2303. doi:10.1128/JVI.02319-10 21177802 PMC3067787

[20] Green TP, Fennell M, Whittaker R, Curwen J, Jacobs V, Allen J, Logie A, Hargreaves J, Hickinson DM, Wilkinson RW, Elvin P, Boyer B, Carragher N, Plé PA, Bermingham A, Holdgate GA, Ward WHJ, Hennequin LF, Davies BR, Costello GF. 2009. Preclinical anticancer activity of the potent, oral Src inhibitor AZD0530. Mol Oncol 3:248–261. doi:10.1016/j.molonc.2009.01.002 19393585 PMC5527863

[21] Wang Q, Xin Q, Shang W, Wan W, Xiao G, Zhang LK. 2021. Activation of the STAT3 signaling pathway by the RNA -dependent RNA polymerase protein of arenavirus. Viruses 13:976. doi:10.3390/v13060976 34070281 PMC8225222

[22] Maldonado-García D, Salgado-Lucio ML, Roa-Espitia AL, Reyes-Miguel T, Hernández-González EO. 2017. Calpain inhibition prevents flotillin re-ordering and Src family activation during capacitation. Cell Tissue Res 369:395–412. doi:10.1007/s00441-017-2591-2 28432466

[23] Riento K, Frick M, Schafer I, Nichols BJ. 2009. Endocytosis of flotillin-1 and flotillin-2 is regulated by Fyn kinase. J Cell Sci 122:912–918. doi:10.1242/jcs.039024 19258392 PMC2871078

[24] Coyne CB, Bergelson JM. 2006. Virus-induced Abl and Fyn kinase signals permit coxsackievirus entry through epithelial tight junctions. Cell 124:119–131. doi:10.1016/j.cell.2005.10.035 16413486

[25] Pereira S, Lowell C. 2003. The Lyn tyrosine kinase negatively regulates neutrophil integrin signaling. J Immunol 171:1319–1327. doi:10.4049/jimmunol.171.3.1319 12874221

[26] Gilfillan AM, Rivera J. 2009. The tyrosine kinase network regulating mast cell activation. Immunol Rev 228:149–169. doi:10.1111/j.1600-065X.2008.00742.x 19290926 PMC2669301

[27] Poe JC, Hasegawa M, Tedder TF. 2001. CD19, CD21, and CD22: multifaceted response regulators of B lymphocyte signal transduction. Int Rev Immunol 20:739–762. doi:10.3109/08830180109045588 11913948

[28] Xu Y, Harder KW, Huntington ND, Hibbs ML, Tarlinton DM. 2005. Lyn tyrosine kinase: accentuating the positive and the negative. Immunity 22:9–18. doi:10.1016/j.immuni.2004.12.004 15664155

[29] Hu C, Priceputu E, Cool M, Chrobak P, Bouchard N, Forestier C, Lowell CA, Bénichou S, Hanna Z, Royal V, Jolicoeur P. 2023. NEF-Induced HIV-associated nephropathy through HCK/LYN tyrosine kinases. Am J Pathol 193:702–724. doi:10.1016/j.ajpath.2023.02.006 36868467 PMC10284032

[30] Li MY, Naik TS, Siu LYL, Acuto O, Spooner E, Wang P, Yang X, Lin Y, Bruzzone R, Ashour J, Evans MJ, Sanyal S. 2020. Lyn kinase regulates egress of flaviviruses in autophagosome-derived organelles. Nat Commun 11:5189. doi:10.1038/s41467-020-19028-w 33060596 PMC7564011

[31] Schlie K, Maisa A, Freiberg F, Groseth A, Strecker T, Garten W. 2010. Viral protein determinants of Lassa virus entry and release from polarized epithelial cells. J Virol 84:3178–3188. doi:10.1128/JVI.02240-09 20071570 PMC2838109

[32] Ahangari F, Becker C, Foster DG, Chioccioli M, Nelson M, Beke K, Wang X, Justet A, Adams T, Readhead B, et al.. 2022. Saracatinib, a selective Src kinase inhibitor, blocks fibrotic responses in preclinical models of pulmonary fibrosis. Am J Respir Crit Care Med 206:1463–1479. doi:10.1164/rccm.202010-3832OC 35998281 PMC9757097

[33] Liu W, Guo TF, Jing ZT, Yang Z, Liu L, Yang YP, Lin X, Tong QY. 2018. Hepatitis B virus core protein promotes hepatocarcinogenesis by enhancing Src expression and activating the Src/PI3K/Akt pathway. The FASEB Journal 32:3033–3046. doi:10.1096/fj.201701144R 29401603

[34] Valencia HJ, de Aguiar MCAM, Costa MA, Mendonça DC, Reis EV, Arias NEC, Drumond BP, Bonjardim CA. 2021. Evaluation of kinase inhibitors as potential therapeutics for flavivirus infections. Arch Virol 166:1433–1438. doi:10.1007/s00705-021-05021-1 33683474 PMC7938686

[35] Shin JS, Jung E, Kim M, Baric RS, Go YY. 2018. Saracatinib inhibits middle east respiratory syndrome-coronavirus replication in vitro. Viruses 10:283. doi:10.3390/v10060283 29795047 PMC6024778

[36] Stefanova D, Olszewski D, Glitscher M, Bauer M, Ferrarese L, Wüst D, Hildt E, Greber UF, Werner S. 2024. FGF receptor kinase inhibitors exhibit broad antiviral activity by targeting Src family kinases. Cell Mol Life Sci 81:471. doi:10.1007/s00018-024-05502-x 39621133 PMC11612106

