# Supplementary material for: Role of Src family kinases Fyn and Lyn in arenavirus infection
Source: J Virol. 2026 Mar 31;100(4):e00241-26. doi: 10.1128/jvi.00241-26 (PMC13098249; doi:10.1128/jvi.00241-26)
Supplement: Supplemental material — Supplemental methods, Fig. S1 to S4, and Tables S1 and S2. [file jvi.00241-26-s0001.docx]

**Supplementary Materials and Methods**

**Reverse Co-IP assay**

A549 cells were transfected with pcDNA3.1-Fyn and pcDNA3.1-Lyn respectively. After 24 hours of serum starvation post transfection, the cells were infected with rLCMV-LASV GP or LCMV Cl13 at an MOI of 1. At 15 min post infection, the cells were harvested and the proteins were extracted with Pierce™ IP Lysis Buffer (Thermo Fisher Scientific). The cell lysates were incubated with anti-Fyn mAb (Cell Signaling Technology; 1:50), anti-Lyn mAb (Cell Signaling Technology; 1:50) and anti-Src mAb (Cell Signaling Technology; 1:50) at 4 ℃ overnight. The lysate and antibody (immune complex) solution was transferred to a test tube containing the pellet of pre-washed magnetic beads, and incubated the mixture with rotation at room temperature for 20 minutes. Then, the magnetic beads were pelleted using a magnetic separation rack. Subsequently, the beads were washed with PBST and the elates were collected with elution buffer. The lysates and eluates were analyzed by western blotting. The primary antibodies were as follows: anti-Fyn mAb (Cell Signaling Technology; 1:1000), anti-Lyn mAb (Cell Signaling Technology; 1:1000) and anti-Src mAb (Cell Signaling Technology; 1:1000), anti-LCMV NP pAb (prepared in this laboratory, 1:1000), anti-p-Tyr mAb (Cell Signaling Technology; 1:1000). The secondary antibodies were as follows: HRP goat anti-mouse IgG, HRP goat anti-rabbit IgG (both from ABclonal, 1:5000). Protein bands were visualized using Enhanced Chemiluminescence (ECL) and a ChemiDoc imager (Bio-Rad Laboratories).

**Immunoblotting assay for SFKs phosphorylation**

Following 24 h of serum starvation, A549 cells were pre-incubated with the drug for 1 h and then placed in an ice bath at 4 ℃ for 30 min to achieve temperature equilibration. Subsequently, the cells were incubated with LCMV (MOI =1) for 1 h to allow virus attachment. A parallel control group was included, which was left untreated with drug under the same temperature conditions. After incubation at 4 ℃, the cells were transferred to a 37 ℃ incubator for 15 min, and cell lysates were then harvested. Protein expression levels were detected by western blotting assay and densitometric analysis was performed using ImageJ software from three independent experiments.

**Gene knockdown (KD) growth kinetics assays**

Transfection was performed using the same protocol as in the preceding siRNA-mediated gene knockdown experiments, and A549 cells were incubated with the transfection mixture at 37 ℃. Cells were lysed at 24 h and 48 h. Gene mRNA and protein levels were verified using qRT‑PCR and Western blotting assays. The primary antibodies were as follows: anti-GAPDH (Abclonal; 1:1000), anti-Fyn (Cell Signaling Technology; 1:1000), anti-Lyn (Cell Signaling Technology; 1:1000) and anti-Ack1 (Abcam; 1:200), anti-Hck (Abcam, 1:200). The secondary antibodies were as follows: HRP goat anti-mouse IgG, HRP goat anti-rabbit IgG (both from ABclonal, 1:5000).


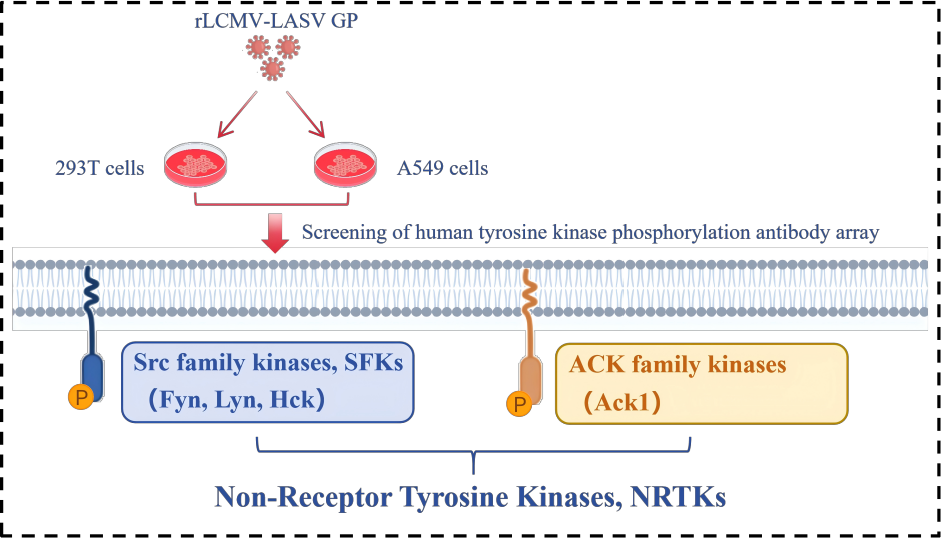


**Figure S1** Schematic diagram illustrating the relationships among kinases activated in both 293T and A549 cells post-infection.


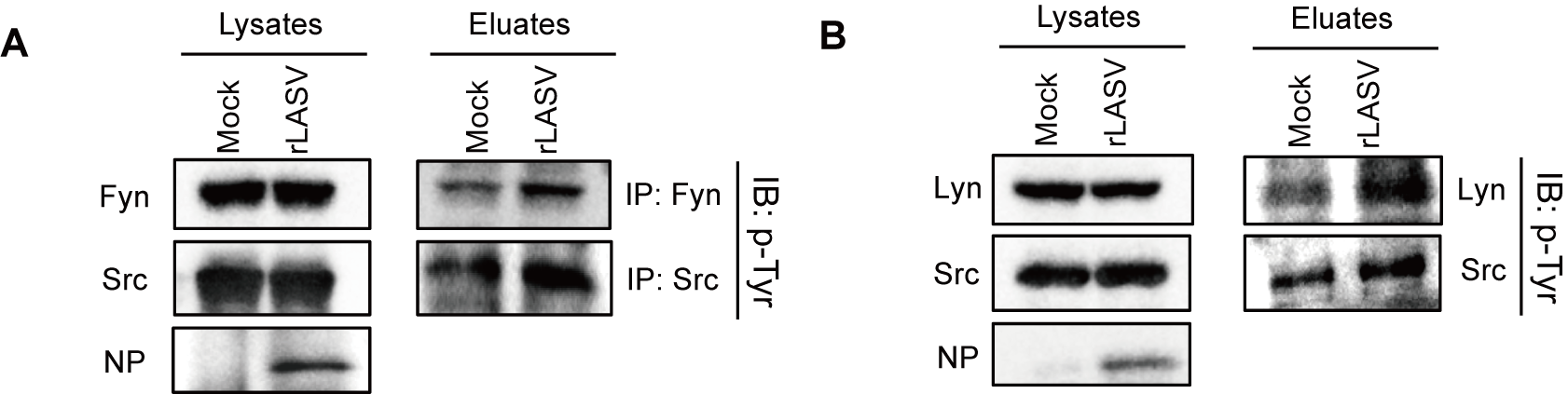


**Figure S2** Analysis of Fyn and Lyn phosphorylation by reverse IP following rLCMV-LASV GP infection. (A) Reverse IP analysis of Fyn phosphorylation post-infection. (B)Reverse IP analysis of Lyn phosphorylation post-infection. A549 cells were transfected with pcDNA3.1-Fyn and pcDNA3.1-Lyn, respectively. The cells starved of serum for 24 hours were infected with rLCMV-LASV GP or LCMV (MOI=1). The cells were harvested 15 minutes after infection. Protein bands were visualized using ECL and a ChemiDoc imager.

**Figure S3** Analysis in SFKs phosphorylation levels in LCMV-infected cells treated with saracatinib. A549 cells were pre-incubated with saracatinib (50 μM) for 1 h, ice-bathed for 30 min, infected with LCMV (MOI=1) at 4 ℃ for 1 h, and further incubated at 37 ℃ for 15 min before sample harvest. Protein bands were detected using western blot assay (A) and analyzed using Image J software (B). Data represent mean ± SD of triplicate experiments and significance was determined by *t*-test; *P<0.05.

**Figure S4** Growth kinetics assays for Ack1, Hck, Fyn, and Lyn knockdown. A549 cells were incubated with the siRNA transfection mixture at 37 ℃, and cells were lysed at 24 h and 48 h. Protein expression levels of Ack1 (A), Hck (C), Fyn (E), and Lyn (G) were detected by western blotting assay. mRNA levels of Ack1 (B), Hck (D), Fyn (F), and Lyn (H) were determined by qRT-PCR assay. Data represent mean ± SD of triplicate experiments.

**Table S1 Sequences of siRNAs used for gene knockdown**

| Name | Sequence5’-3’ | Usage |
| --- | --- | --- |
| ACK1-1 | GCCUGUCCCACUUUGAGUATT | gene knockdown |
| ACK1-2 | GCAAGUCGUGGAUGAGUAATT | gene knockdown |
| ACK1-3 | GCUGGGUGAUGGUUCCUUUTT | gene knockdown |
| Hck-1 | GUCGGAGGCAAUACAUUCUTT | gene knockdown |
| Hck-2 | CACUAAAGGAAGCUACUCUTT | gene knockdown |
| Hck-3 | GGAUGUCAAACCCUGAAGUTT | gene knockdown |
| Fyn-1 | GCGAGAGUGAAACCACCAATT | gene knockdown |
| Fyn-2 | GGUUACAUUCCCAGCAAUUTT | gene knockdown |
| Fyn-3 | GGCCCAGUUUGAAACACUUTT | gene knockdown |

**Table S2 Sequences of primers used for qRT-PCR**

| Name | Sequence5’-3’ | Usage |
| --- | --- | --- |
| ACK1-F | CGCCCATGAAGATGGTGACA | qRT-PCR |
| ACK1-R | AGAAGGTGCGTGTCTTCAGG | qRT-PCR |
| Hck-F | TCCCACATCCACCATCAAGC | qRT-PCR |
| Hck-R | CCACTCCCCGGATTCCTCTA | qRT-PCR |
| Fyn-F | TGATCTAAACGTGGAAAAAGACCAG | qRT-PCR |
| Fyn-R | GGTCCCCGTATGAGACGAAG | qRT-PCR |
| Lyn-F | ATAAACAGCAAAGGCCAGTTCCA | qRT-PCR |
| Lyn-R | AAAGACAAGTCGTCCGGGTG | qRT-PCR |
| LCMV NP-F | AAGCTGAAGGCCAAGATCAT | qRT-PCR |
| LCMV NP-R | GAGGCTTTCTCATCCCAACTAT | qRT-PCR |
